# Long-term moderate wind induced sediment resuspension meeting phosphorus demand of phytoplankton in the large shallow eutrophic Lake Taihu

**DOI:** 10.1371/journal.pone.0173477

**Published:** 2017-03-16

**Authors:** Jian-Ying Chao, Yi-Min Zhang, Ming Kong, Wei Zhuang, Long-Mian Wang, Ke-Qiang Shao, Guang Gao

**Affiliations:** 1 State Key Laboratory of Lake Science and Environment, Nanjing Institute of Geography and Limnology, Chinese Academy of Sciences, Nanjing, Jiangsu Provence, China; 2 Nanjing Institute of Environmental Sciences, Ministry of Environmental Protection, Nanjing, Jiangsu Provence, China; 3 University of Chinese Academy of Sciences, Beijing, China; University of Hyogo, JAPAN

## Abstract

The objective of this study was to investigate the impact of sediment resuspension and phosphorus (P) release on phytoplankton growth under different kinds of wind-wave disturbance conditions in the large and shallow eutrophic Lake Taihu in China. Short-term strong wind (STSW) conditions, long-term moderate wind (LTMW) conditions, and static/calm conditions were investigated. To address this objective, we (1) monitored changes in surface water P composition during field-based sediment resuspension caused by STSW conditions in Lake Taihu, and also conducted (2) a series of laboratory-based sediment resuspension experiments to simulate LTMW and calm conditions. The results showed that under both strong and moderate wind-wave conditions, suspended solids (SS) and total phosphorus (TP) in the water column increased significantly, but total dissolved phosphorus (TDP) and soluble reactive phosphorus (SRP) remained low throughout the experiments, indicating that the P released from sediments mainly existed in particulate forms. In STSW conditions, alkaline phosphatase activity (APA) and enzymatically hydrolysable phosphorus (EHP) increased rapidly, with the peak value occurring following the peak value of wind speed for 1–2 days, and then rapidly decreased after the wind stopped. Under LTMW conditions, APA and EHP increased steadily, and by the end of the laboratory experiments, APA increased by 11 times and EHP increased by 5 times. Chlorophyll a (Chl-a) in LTMW conditions increased significantly, but remained low under STSW conditions, demonstrating that the former type of sediment P release promoted phytoplankton growth more effectively, and the latter type did not. Despite the fact that STSW conditions resulted in the release of more TP, TP settled to the bottom rapidly with SS after the wind stopped, and did not promote algal growth. Under LTMW conditions, suspended particulate P was hydrolyzed to SRP by phosphatase and promoted algae growth. Algal growth in turn secreted more phosphatase and accelerated particulate P regeneration, which may be the main mechanism of sediment bio-available P release that promotes phytoplankton growth in shallow lakes.

## Introduction

Phosphorus (P) is generally considered the main limiting nutrient for growth of phytoplankton in freshwater systems, and excessive imports of P cause eutrophication [1]. The release of P from sediments is an important nutrient source that can induce continuous eutrophication in lakes even if external inputs are reduced [2, 3]. The release of P from sediments in shallow lakes mainly occurs under static or dynamic conditions [4, 5], accompanied by a large amount of P release, playing a vital role in P cycling and promoting the growth of phytoplankton and subsequent phytoplankton blooms in the water column [6]. For example, research shows that the flux of total P annually released by sediments in Lake Taihu is 21,000 tons, which is approximately 2–6 times the P input from external sources [5]. Static release is the main internal nutrient release mechanism in deep lakes and small shallow lakes and is greatly affected by such factors as sediment redox conditions [7] and pH of the water column [8, 9]. In large shallow lakes, due to frequent and intense wind-induced hydrodynamic disturbances, dynamic release is the main type of internal release [6, 10, 11]. For example, research showed that 88% of total P internal release in Lake Taihu occurs under dynamic conditions [4], and lake sediment resuspension accounts for 85% of the vertical flux of resuspended particulates [12].

Although wind-wave disturbances cause a large amount of P release from sediments, not all resuspended P can be converted into soluble reactive phosphorus (SRP) that can be directly assimilated by algae. The impacts of sediment resuspension caused by different hydrodynamic disturbances, especially STSW and LTMW conditions, on the P geochemical cycle in shallow lakes and its ecological effects remain unclear. For example, continual monitoring of STSW disturbance processes showed that the amount of resuspended sediments and total P increased during the experiment, but SRP and total dissolved P (TDP), which are more important than particulate nutrients, did not increase [6, 11]. The amount of TP quickly decreased to the pre-disturbance level as the disturbance stops [13]. Other research argues that sediment resuspension resulted in more efficient removal of dissolved inorganic P from overlying water due to strong oxidation and a higher contact probability between P and P reactants, such as crystalline iron, aluminum oxides, and organic matter, as a result of sediment resuspension and a resultant decreased in the bioavailability of suspended P particulates [14]. Research on the other hand shows that lake sediment features a high amount of organic P, for example, the percentage of organic P in lake sediments is 11.5% in Lake Meiliang Bay in Lake Taihu, and 43.6% in Lake Hongfeng in Southwestern China Plateau region[15]; Relative contribution of organic P in Lake Kasumigaura in Japan was 25.6% (ranged from 22.6% to 30.0%) in sedimens, and 74.3% (ranged from 72.8% to 80.8%) in suspended particles [16]. Organic P is fragile to be decomposition and mineralization [17, 18]. Mineralization of organic P in suspended particulates is considered the main source of internal P loading [19] and resuspended sediments may serve as a potential source of nutrients for phytoplankton growth and reproduction. However, the above research on P release from sediments during hydrodynamic disturbances has contrary conclusions, results are limited to nutrient release, and the relationship between hydrodynamic disturbance and phytoplankton growth is unclear.

In this study, we conducted field observations of STSW conditions, laboratory experiments of LTMW conditions and calm/static conditions to investigate the P release process, organic P degradation dynamics and phytoplankton growth, in order to assay the impact of different wind-wave conditions on P release and phytoplankton growth in Lake Taihu.

## Materials and methods

### Study area

**Fig 1:**
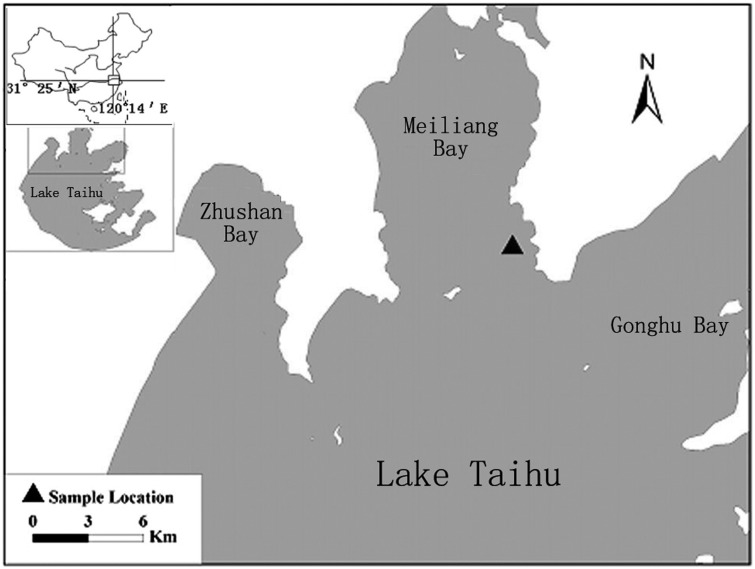
Map showing the location of Lake Taihu and ▲ indicates the field-based observation point.

Lake Taihu, located in eastern China, near the lower reaches of Yangtze River, is the third largest freshwater lake in China, and is an important drinking water, fishing, and tourism resource for Jiangsu Province. It has a water surface area of 2427.8 km^2^ and an average water depth of 1.9 m. The annual average air temperature is 14.9–16.2°C and the climate is a SE-NW monsoon climate. Recent toxic cyanobacterial blooms caused by excessive human nutrient loads have focused attention on controlling blooms and restoring the lake to acceptable water quality nutrient conditions. Both field-based observation and collection of materials for simulated experiments were performed in Meiliang Bay, which is a typical phytoplankton-dominated lake area that is located in the northern part of Lake Taihu (Fig 1)[20, 21].

### Field observation and sampling of STSW conditions

It is thought that the critical wind speeds at which sediments become suspended is 2 m·s^-1^, and become intensively suspended is 6 m·s^-1^ in Lake Taihu [5, 22, 23]. Thus, we classified wind-wave conditions in Lake Taihu into three types according to wind speed: calm conditions (wind speed: < 2 m·s^-1^), moderate wind conditions (wind speed: 2~6 m·s^-1^) and strong wind conditions (wind speed: > 6 m·s^-1^), and each type of wind speed occurred at frequencies of 12%, 82% and 6% of the year, respectively.

**Fig 2:**
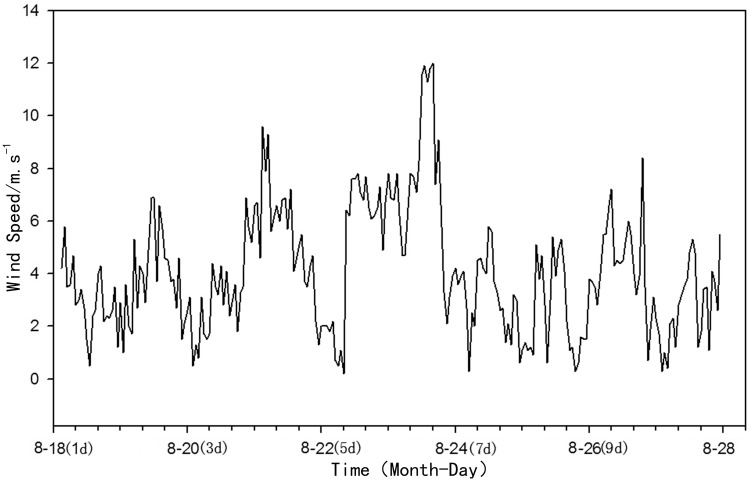
Ten minute averaged wind speed in the observation point during STSW conditions.

In this study, we observed strong wind conditions in the field from August 18–27, 2014 (Fig 2). The location of the field observation is near the east bank of Meiliang Bay of Lake Taihu (120°12′49.67″E, 31°29′09.99″N; Fig 1), approximately 200 m from the bank and 1.6m in depth, and the wind velocity indicator was 10 meters higher than the lake surface. The authority of field experiment location is Wuxi water conservancy bureau, Jiangsu Provence, and there is no specific permission required for the location. The field studies did not involve endangered or protected species. The surface water samples were taken once a day at 14:00. Surface water samples (typically 0.5 m) were collected with 5L carboys once a day at 14:00 and three parallel water samples were simultaneously taken each time. During each sampling event, water temperature (WT), dissolved oxygen (DO), pH, water clarity (secchi depth, SD) and electrical conductivity (EC) were also recorded with a continuously recording multiparameter underwater sensor (YSI 6600, Yellow Springs Instruments). Before deployment, sensors were calibrated and accuracy was checked by measuring standards with an average error of 2%.

### Laboratory experiments of LTMW conditions

**Fig 3:**
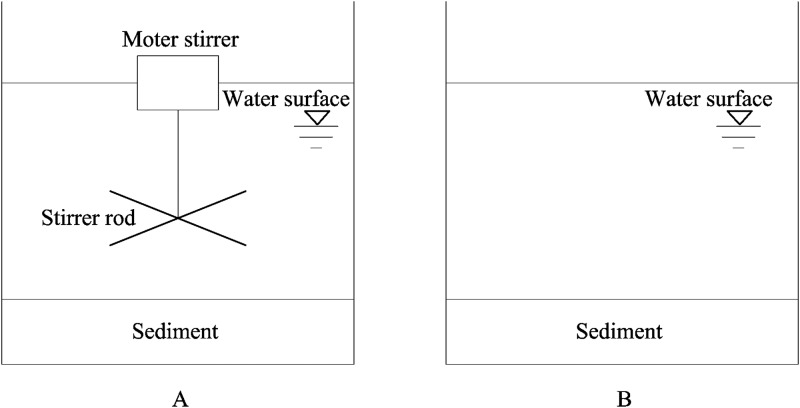
Laboratory experimental device for simulated LTMW turbulence-induced sediment resuspension (A) and static release (B). The water and sediment were collected from the same location of field observations of the STSW disturbance, which is shown in Fig 1. Sediment thickness was 5cm and water depth was 40cm. The stirrer rod was 5 cm under the water surface.

The simulation experiment of moderate wind conditions was performed in indoor 60L glass tanks (40cm*30cm*50cm). Sediment and lake water used in the experiment were collected from the same site where the in situ experiment was performed and was then placed in the simulated system. First, mixed surface sediments from 0–10 cm in Lake Taihu were collected using a Petersen grab sampler, as common estimates of the active sediment depth varies between a thin surface to the top 10 cm in shallow lakes [24]. Sediment was immediately brought back to the laboratory and placed on the bottom of glass tanks, with the sediments ~5 cm in thickness. Next, lake water was gently added along the tank wall to a depth of 40cm (avoiding disturbing the sediment as much as possible). The experiment started after suspended particulates in the water column in the tanks fully settled (standing statically for 48 hours). Prior to the experiment, sediment was sieved (mesh size: 1.7 mm) to remove larger invertebrates, mixed to ensure heterogeneity, and analyzed for some basic chemical parameters. The water was filtered through a 64-um mesh plankton net to remove larger zooplankton and seston particles. Two treatments were applied—one treatment with moderate disturbances and a control treatment with no disturbance, which respectively represent the disturbance conditions caused by continual moderate winds and static or calm conditions in lakes. Both treatments were performed in three parallel tests. In order to simulate moderate wind-wave conditions, we employed a motor stirrer to mimic natural disturbance (Fig 3). According to previous studies, the correlation between SS and wind speed exactly filed the exponential function [23, 25, 26]. Based on the results obtained from several preliminary runs and suspended solid concentrations under moderate wind-wave conditions in Lake Taihu, a blade stirrer was inserted at the water surface for 5cm, operated at 80 rad min^-1^. Water samples were taken respectively on the 1^st^, 2^nd^, 4^th^, 6^th^, 9^th^, 12^th^, and 15^th^ days after the experiment began, and a 200-ml water sample was collected each time. WT, DO, pH were also recorded with a portable DO meter (HACH HQ30d, USA).

### Analytical methods

The water samples collected both in the field observation and laboratory experiments were analyzed for SS, particulate organic matter (POM), total phosphorus (TP), total dissolved phosphorus (TDP), soluble reactive phosphorus (SRP), alkaline phosphatase activity (APA), enzymatically hydrolysable phosphorus (EHP), and chlorophyll a (Chl-a). SRP concentrations were determined spectrophotometrically, according to the molybdenum blue method [27]. TP and TDP concentrations were determined after thawing with the use of combined persulfate digestion [28], followed by spectrophotometric analysis as for SRP. Chl-a concentrations were determined spectrophotometrically after extraction in 90% hot ethanol [29]. APA was assayed by an ultraviolet-visible spectrophotometer as the release of p-nitrophenol from model substrate p-nitrophenyl phosphate according to Paul et al. [30]. EHP in the water column was measured using the method of natural hydrolysis of organic P by enzymes in the lake water [31]. EHP refers to the total quantity of P in various forms in the water column that can be converted into SRP in a short time (5 days).

The physicochemical properties of sediments used for laboratory experiments were also analyzed. The pH of sediment samples were determined on sediments suspended in deionized water at a sediment: water ratio of 1:2.5 [32]. Organic matter (OM) in sediments was determined by loss of ignition at 550℃ for 4h [33]. Total P (TP) in sediments was determined by 1mol L^-1^ HCl extraction (16h) after pretreatment for 2h at 500°C [34]. Particle size distribution of sediments was analyzed using a laser particle analyzer [2].

The wind speed data came from wind speed observations of the Taihu Laboratory for Lake Ecosystem Research, Chinese Academy of Sciences. The height of the wind recorder is about 10 meters higher than the lake surface.

#### 2.5 Data analysis

For each experimental run, the measured values in all trials for a given parameter were pooled to formulate a single dataset, which in turn was used to compute the mean and standard deviation for that parameter. The datasets for all experimental runs for each parameter were then used to conduct a multiple comparison t test in SPSS 13.0. The average values, along with maximum and minimum deviations, were plotted against measurement day in Sigmaplot 9.0 to visually examine differences.

## Results

### Variation in particulate matter in different disturbance types

During the in situ observation, we observed a complete strong wind-wave disturbance process, in which the wind speed underwent a low-high-low process. The wind speed data is listed in Fig 2. Between the 1^st^ and 3^rd^ day of the experiment, wind speed was relatively slow, averaging 3 m·s^-1^. Between the 4^th^ and 6^th^ day, a strong wind occurred, with speed averaging 5.4 m·s^-1^ on the 5^th^ day (Aug 22), and 7.4 m·s^-1^ on the 6^th^ day. The average wind speed between 12:00 and 16:00 was 11.72 m·s^-1^ (Aug 23). In the last 4 days (between the 7^th^ and 10^th^ days), wind speed decreased, averaging 2.9 m·s^-1^. In the first 3 days when the wind wave was relatively small, SS ranged from 13.2 to 21.9 mg·L^-1^. As wind speed gradually increased on the 4^th^ day, SS increased quickly to 29.3 mg·L^-1^ and finally reached 251 mg·L^-1^ on the 5^th^ day, which was 8.6 times higher than that on the previous day. On the 6^th^ day, the wind speed reached the maximum value in the experimental period, and SS in the water column accordingly reached a peak value of 325.5 mg·L^-1^, which was 1.29 times that of SS on the previous day and 15.7 times that of the average value during the calm period. When the strong wind ended, SS quickly decreased to 84 mg·L^-1^ on the 7^th^ day (August 24) and 30.9 mg·L^-1^ on the 8^th^ day (August 25), which was slightly higher than the level before the wind occurred. The above observation results showed that wind-wave disturbance was the main factor that caused intensive resuspension of sediment in the water column, and suspended particulates that increase due to strong wind-wave disturbance will quickly decrease with wind speed. The average wind speed had a significant positive correlation with SS in the water column (r = 0.735, p<0.05). As SS increased, POM in suspended particulates gradually increased as wind speed increased, reaching a maximum of 23.0mg·L^-1^ on August 23 when the wind speed was highest, and then decreased. This pattern was very similar to that of SS. However, the percentage of POM in SS had an opposite trend; that is, as wind speed increased, the POM/SS ratio gradually decreased, reaching a minimum of 7.1% on August 23 (Table 1), which indicates that numerous inorganic particulates in the sediment entered the water column as a result of the wind-wave conditions, as the organic matter content in sediment was only 6.52% (Table 2).

**Table 1:** Physical and chemical properties of lake water for STSW observations.

Water Parameters	1d	2d	3d	4d	5d	6d	7d	8d	9d	10d	Average
Wind speed m/s	3.1	3.6	3.3	5.4	4.9	7.4	2.9	2.9	4.6	3	4.1
DO /mg O_2_·L^-1^	10.82	10.85	10.91	10.77	11.23	11.16	10.94	11.08	10.85	10.81	10.94
WT /℃	31.5	31.7	31.4	30.7	30.5	30.6	30.9	31.2	31.0	31.2	31.1
pH	8.24	8.33	8.67	8.22	8.04	8.56	8.20	8.22	8.28	8.14	8.29
SD /m	0.22	0.25	0.25	0.15	0.10	0.12	0.18	0.22	0.25	0.03	0.18
Chl-a /μg·L^-1^	65.84	55.80	62.25	37.16	21.30	43.51	42.18	18.72	38.17	37.16	42.21
TP /mg·L^-1^	0.088	0.103	0.085	0.094	0.160	0.242	0.114	0.105	0.122	0.100	0.121
TDP /mg·L^-1^	0.079	0.085	0.070	0.074	0.070	0.073	0.067	0.080	0.060	0.066	0.072
SRP /mg·L^-1^	0.027	0.036	0.023	0.017	0.021	0.036	0.040	0.040	0.034	0.036	0.031
SS /mg·L^-1^	18.6	21.9	13.1	29.3	251.0	325.5	84.0	30.9	90.3	33.1	89.8
POM /mg·L^-1^	4.1	4.0	3.3	4.6	18.4	23.0	10.9	6.4	6.9	4.3	8.6
POM /%	21.9	18.1	25.1	15.6	7.3	7.1	13.0	20.7	11.5	13.0	15.3

DO, WT, SD, Chl-a, TP, TDP, SRP, SS, POM, POM% represent dissolved oxygen, temperature, secchi depth, chlorophyll a, total phosphorus, total dissolved phosphorus, soluble reactive phosphorus, SS, particulate organic matter, and percentage of POM/SS.

**Table 2:** Physiochemical properties of sediments in Meiliang Bay.

Characteristics	Values
DW (%)	79.3
POM (%)	6.52
pH	7.8
TP(mg.kg-1)	745
Particle size distribution	
Clay(<4um,%)	12.5
Slit(4~63um,%)	74.4
Sand(>63um,%)	13.1

**Fig 4:**
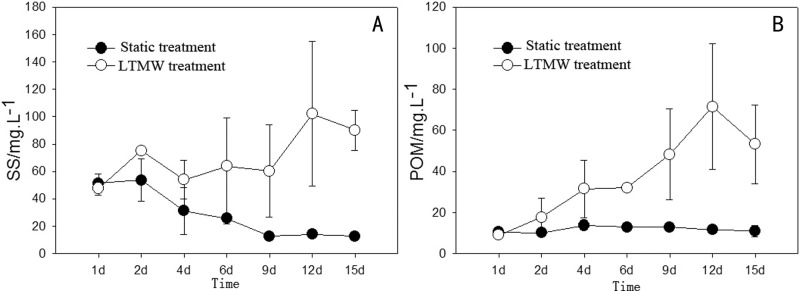
Changes of SS (a) and POM (b) concentrations in LTMW and static treatments.

SS and POM concentration in the water column during the LTMW disturbance experiment are shown in Fig 4. The minimum value of suspended solids in the disturbance group was 47.67 mg·L^-1^, which occurred at the beginning of the experiment, the maximum value was 102 mg·L^-1^, which occurred on the 12^th^ day, and the average value was 70.43 mg·L^-1^. In the indoor simulation experiment of moderate disturbance, due to control of the disturbance intensity, SS in the water column remained between 50 and 100 mg·L^-1^. Complete sediment suspension did not occur, as the integrity of the sediment-water interface was maintained, and the relationship between disturbance intensity and SS was similar to that in moderate wind conditions in Lake Taihu [22, 23]. The amount of POM in the water column increased progressively in the first 12 days of the experiment, increasing from 9 mg·L^-1^ at the beginning of the experiment to 71.57 mg·L^-1^ on the 12^th^ day. In the entire experimental period, POM accounted for 53% of the total suspended particulates. In the static treatment group, SS and POM do not change obviously, remaining at low levels during the experiment.

### Changes in P concentration and composition in water column under different disturbance types

Variations in the concentration of P in all forms in the water column during the in situ strong wind-wave disturbance experiment are shown in Table 1. The trend of total P concentration was similar to that of wind speed and SS, with correlation coefficients of *r*_*wind speed*_ = 0.786 (*n* = 30, *p*<0.01) and r_ss_ = 0.953(*n* = 30, *p*<0.01). When the wind speed increased on August 22, total P in the water column quickly rose and reached a maximum value of 0.242 mg·L^-1^ on August 23, which is 2.5 times that of the total P concentration in the calm period during the first 3 days. After the strong wind ended on Aug. 24, total P obviously decreased. However, in the entire experimental period, TDP in the water column did not change obviously, remaining ~0.07 mg·L^-1^ and averaging 0.072 mg·L^-1^. SRP increased slightly in the strong wind period, and the maximum value of SRP of 0.04 mg·L^-1^ occurred on the 6^th^ and 7^th^ day in the experiment, which was 1–2 days after the strong wind period. Later, SRP decreased, lagging 1–2 days behind the SS and TP peak values. TDP did not change obviously and SRP increased slightly, which was significantly different from that of TP. So the SRP/TDP ratio increased from 30%-40% at the beginning of the experiment to 50%-60% at the end of the experiment.

**Fig 5:**
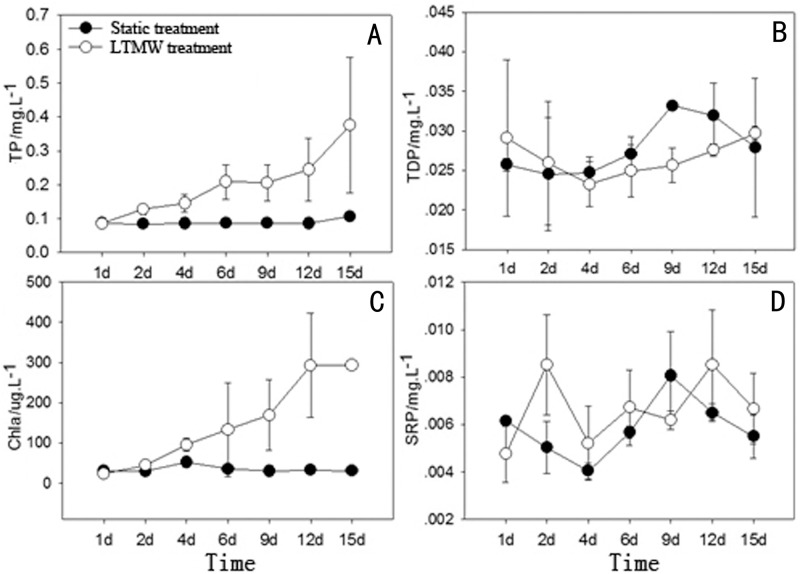
Changes of TP, TDP, SRP and Chl-a concentrations in LTMW and static treatments.

Variations in the concentration of P of all forms in laboratory experiments of LTMW disturbance are shown in Fig 5. TP gradually increased from 0.085 mg·L^-1^ at the beginning to 0.376 mg/L at the end of the experiment, averaging 0.199 mg·L^-1^, which was significantly higher than that of the static treatment (0.089mg.L^-1^) (p<0.05, paired samples test, n = 21). TDP and SRP did not show an obvious increase, but fluctuated within certain ranges. TDP ranged from 0.023 to 0.03 mg·L^-1^, averaging 0.027 mg·L^-1^, and SRP ranged from 0.0047 to 0.0085 mg·L^-1^, averaging 0.0066 mg/L. In addition, TDP and SRP in the LTMW treatment were not significantly different from those of static treatment.

### P release dynamics and ecological effects on phytoplankton growth under different disturbance processes

**Fig 6:**
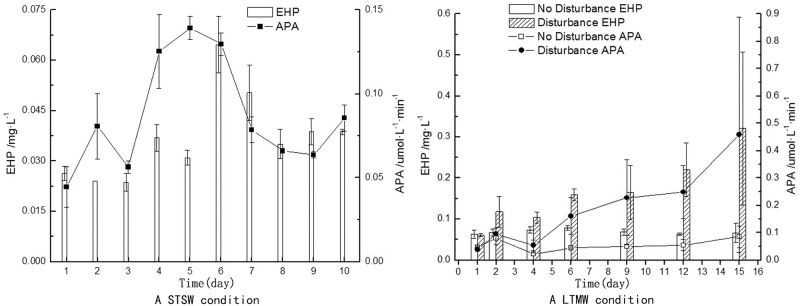
Changes of APA and EHP under different hydrodynamic disturbance types. A represents STSW disturbance conditions and B represents LTMW disturbance conditions.

Under STSW conditions, EHP and APA in the water column increased obviously (Fig 6a), and the rise in the latter was particularly significant. During the experiment, APA ranged from 45 to 139 nmol·L^-1^·min^-1^, averaging 87 nmol·L^-1^·min^-1^. Specifically, in the first three days (calm period) of the experimental period, APA was at a low level, averaging 61 nmol·L^-1^·min^-1^, and in the three days of high wind speed, APA averaged 131 nmol·L^-1^·min^-1^, with a peak value of 139 nmol·L^-1^·min^-1^, which was the highest in the experimental period. After the wind died, APA quickly decreased to 73 nmol·L^-1^·min^-1^, and finally returned to the average level of the calm period. EHP concentration also increased with wind speed, with an average value 0.037 mg.L^-1^, and the highest value occurred on the 6^th^ day, with a value of 0.065 mg.L^-1^. During the experiment, the proportion of EHP in TP in the water column averaged 30.5%, decreasing with increasing wind speed, with the maximum proportion of 44.2% occurring on the 7^th^ day (August 24). Under STSW conditions, Chl-a in the water column was low, with an average value of only 42.21 μg·L^-1^ (Table 2). Chl-a concentration showed a slight decrease with wind; in the calm period of the first 3 days, average Chl-a was 61.30 μg·L^-1^ and during strong winds the average concentration was 33.99μg·L^-1^. Throughout the wind event, the maximum value was 65.84 μg·L^-1^, which appeared on the first day of the experiments, and the minimum value was only 18.72 μg·L^-1^.

In the LTMW laboratory experiments, both EHP and APA continually increased (Fig 6b). In the experimental period, EHP ranged from 0.061 to 0.321 mg·L^-1^, averaging 0.164 mg·L^-1^, which is 4 times than that of STRW conditions. However, EHP in static treatments did increase, ranging from 0.063 to 0.078 mg·L^-1^ and averaging 0.069 mg·L^-1^. EHP in the disturbance treatment were significantly higher that of the static treatment (p<0.01, n = 21), and the average EHP/TP ratio was 82.4%, which was also significantly higher than that in STSW conditions. Trends in APA were similar to that of EHP. As the disturbance continued, APA in the water column increased from 39 nmol·L^-1^·min^-1^ at the beginning of the experiment to 458 nmol·L^-1^·min^-1^ at the end of the experiment, averaging 184 nmol·L^-1^·min^-1^, which was significantly higher than that of the static treatment (p<0.01, n = 21). In the LTMW treatment, Chl-a concentration increased (Fig 5c), rising from 23.8 μg·L^-1^ at the beginning to 293.1 μg·L^-1^ at the end of the experiment, increasing by more than 11 times, and was significantly correlated with suspended solids, POM and total P (p<0.05, n = 21). Chl-a concentration in the water column of the static treatments remained at a low level of ~30 μg·L^-1^. The above data showed that algal primary productivity in the LTMW treatment significantly increased, while algal biomass did not increase under static and STSW conditions.

## Discussion

There are several P transport mechanisms from sediments to overlaying waters, such as wind-induced water disturbance, diffusion, and bioturbation [35]. In Lake Taihu, the first two are the main types of P release [5].Results of this study have shown that both long-term moderate and STSW induced P release were more intensive than under calm conditions, as TP, TDP and SRP concentrations in water column of the static treatment remained low, and the two disturbed treatments significantly increased significantly. The result indicated that P release was very small in calm condition via diffusion in Lake Taihu. These findings can also confirm the conclusions that dynamic release of P is 2 order of magnitudes higher than that of static release observed by Qin [5].

This work demonstrated that under STSW conditions, the concentration of SS, POM and TP significantly rose quickly, and the maximum value was multiplicative of that of the calm period. EHP also multiplied, but the EHP/TP ratio peak appeared one day later than that of the EHP peak, which is possibly due to strong wind-wave disturbance causing resuspension of inorganic sediment, which has poor bio-availability and lowered the proportion of EHP as a whole. When the strong wind ended, the rapid settlement of inorganic particulate matter such as sands may be the reason that the EHP/TP ratio peak appeared on the 7^th^ day. The rise of total particulate EHP indicates that sediment suspension results in the increase of bioavailable P for phytoplankton. Meanwhile, APA concentration also increased and peaked on the 5^th^ day.

It is generally believed that a rise in APA indicates insufficient phosphate for algae in the water column, and phosphatase secreted by phytoplankton can decompose organic P and cause the release of SRP from organic matter. However, in the STSW observation, Chl-a concentration remained low, indicating that APA increase may not be attributed to the release from phytoplankton, but from sediment resuspension, for sediment is also a phosphatase resource for the water column [36]. The peak value of APA appeared on the 5^th^ day, which is earlier than SS and TP, perhaps indicating that APA mainly came from the surface layer of sediment, for sediment APA profiles follow a downward decreasing trend [36]. During wind events, APA increased first, reaching a peak value on the 5^th^ day, TP and EHP reached a peak value on the 6^th^ day, and SRP reached a peak value on the 7^th^ and 8^th^ days. The above experimental results indicate that as wind speeds increased during the 4^th^ to 6^th^ days of observation, APA and TP in sediment were resuspended in the water column, adding APA and EHP to the water column. The dissolved nutrients may also be resuspended to the water column with pore water, but we did not observed an increase of TDP and SRP during 4^th^ to 6^th^ days, which may be caused by adsorption of SS, as surface sediments are often oxidized in large shallow lakes, and can absorb dissolved P from water when resuspended. This phenomenon has also been observed previously [37, 38].

The hydrolysis of EHP may result in the release of SRP on the 6^th^ and 7^th^ days, but SRP did not increase significantly. These findings were similar to lab experiments and previous result. Previous research showed that wind-wave disturbances can cause rapid sediment resuspension, leading to a fast increase in SS, TN, and TP in the water column, but will not result in a significant increase in dissolved P, such as TDP and SRP [39]. Some other studies have shown that SRP in the initial disturbance stage (within 1h) increases and then quickly decreases [40], which may be the result of adsorption of PO_4_-P by suspended particulates or colloids, which is similar to this work. Although the rise in SRP was observed during the experiment, Chl-a did not increase significantly, which is possibly attributable to the following two factors.

Firstly, high SS and turbidity in STSW conditions result a low transparency, algae is mixed in the water column homogeneously due to turbulence, and light conditions cannot sustain high algae biomass and also limit algae growth [41, 42]. So even if algae can assimilate P very fast[43], the biomass cannot increase much in STSW conditions. Secondly, there is a starting time which takes several days for algae transform from lag phase to logarithmic phase when algae begin rapid breeding. But the nutrient sufficient condition caused by strong wind only continue several days, and after the strong wind ended, nutrient concentrations decreased to the level of the calm conditions quickly. The results indicate that the disturbance of STSW caused a large amount of particulate P, EHP and APA release from sediments, but the low transparency of the water column and the disturbance conditions in STSW were not suitable for phytoplankton growth and reproduction.

Under LTMW conditions, SS in the water column did not increase quickly, but instead slowly increased; the sediment was not completely resuspended, with only 0.5–1 cm sediment on the surface layer being affected by the disturbance. Results showed that resuspension of a small amount of sediment occurred and SS and TP rose slightly in the LTMW treatment. Although dissolved P concentrations did not increase, P cycling rate and bio-available P increased, and algae largely grew and reproduced.

The results indicate that wind-wave disturbances cannot change SRP, which may be directly used by algae, but caused an increase in particulate P and bioavailable P.

On the other hand, phytoplankton, due to being limited by P, is likely to secrete a large amount of alkaline phosphatase to degrade organic P in suspended particulate matters [44]. The two processes, uptake of SRP by phytoplankton and release of SRP from particulate matters by hydrolysis, keep SRP levels low.

Many studies have shown that particulate organic matter (POM) is rich in nutrients and is an important source of bioavailable nutrients in the water column [45]. Chl-a increasing in the LTMW treatment indicates that phytoplankton biomass increased several times that of the static treatment, and the correlations of Chl-a with SS and TP also indicate algal growth is the primary contribution to the rise of SS and TP in the water. LTMW is a normal condition in Lake Taihu and the annual duration of moderate wind accounts for 82% of the year [5]. The degradation of organic P in POM and release of SRP are possibly the main sources of nutrients necessary for growth of phytoplankton [46]. Therefore, the hydrolysis of suspended particulate organic P and release of SRP may be the main transport mechanisms of P from sediments to overlying water, and the primary method of promotion of phytoplankton growth by sediment resuspension in Lake Taihu.

In the static release experiment, all forms of P in the water column remained at low levels, which indicate that the amount of P static release is very small. The low concentration of bioavailable P in the water column could not meet the growth demand of phytoplankton, for Chl-a concentration was also very low. It is believed that P release occurred in both aerobic and anoxic conditions, and it is generally recognized that P release in an anoxic environment is much greater than that in an aerobic environment, thus leading to diffusion of dissolved P in the sediment pore water to the water column [47, 48]. However, the dissolved oxygen on the water-sediment interface in shallow lakes is relatively sufficient, which is a disadvantage for static diffusion of nutrients to the overlying water [49].

Based on the above analysis, in calm conditions, the static release amount is relatively low, and cannot promote growth of algae. In LTMW wave disturbance conditions, sediment resuspension causes an increase in bio-available P in the water column, and phosphatase released from algae promotes the conversion of bio-available P to SRP. Moreover, phytoplankton growth transforms SRP to particulate organic P, and phytoplankton-derived particulate matter is believed to be more fragile than sediment particles [50]. Thus, bioavailable P accumulates in the water column, and promotes phytoplankton growth by internal P cycling in the water column. The STSW wave disturbance will cause the release of large amounts of P from sediments, multiplication of bio-available P in the water column, and release of large amounts of P in the dissolved state in the pore water. However, because of the short duration of STSW disturbance, suspended particles quickly settle to the bottom after the wind stops, and, because dissolved P will be adsorbed easily by suspended particles, STSW conditions have only a small effect on the growth of phytoplankton.

## Conclusions

This study has revealed the impact of sediment resuspension and P release on phytoplankton growth under different wind-wave disturbance conditions in the large and shallow eutrophic Lake Taihu. Our field and laboratory study showed that:

1. STSW disturbances caused the resuspension of large amounts of sediment, which significantly increased SS, TP, bio-available P and APA in the water column, but had small impacts on SRP and TDP. However, due to the short duration of STSW conditions and low transparency of the water column, the P release from sediment in STSW conditions did not promote phytoplankton growth.
2. LTMW disturbances caused sediment resuspension, which is smaller and more moderate than that of STSW disturbances, and the suspension duration is longer. LTMW conditions also caused increases in SS, TP and bio-available P in the water column. SRP and TDP did not rise significantly, but increase in APA and organic P degradation rate significantly promoted phytoplankton growth.
3. The ecological effect of sediment resuspension on phytoplankton is likely due to the mineralization of resuspended particulate organic P and the release of SRP, and phytoplankton growth also accelerates the mineralization rate by secreting phosphatase. Long-term moderate wind can maintain suspended particulate matter in a suitable concentration in the water column for phytoplankton growth.
